# Efficacy and Safety of Electro-Thumbtack Needle Therapy for Patients With Chronic Neck Pain: Protocol for a Randomized, Sham-Controlled Trial

**DOI:** 10.3389/fmed.2022.872362

**Published:** 2022-04-29

**Authors:** Hangyu Shi, Xinlu Wang, Yan Yan, Lili Zhu, Yu Chen, Shuai Gao, Zhishun Liu

**Affiliations:** ^1^Department of Acupuncture, Guang'anmen Hospital, China Academy of Chinese Medical Sciences, Beijing, China; ^2^Beijing University of Chinese Medicine, Beijing, China; ^3^New Zealand College of Chinese Medicine, Auckland, New Zealand

**Keywords:** acupuncture, chronic neck pain, RCT, electro-thumbtack needle, efficacy and safety

## Abstract

**Background:**

Chronic neck pain is a prevalent condition adversely impacting patients' wellbeing in both life and work experience. Electro-thumbtack needle (ETN) therapy, combining acupuncture with transcutaneous stimulation, might be one of the effective complementary and alternative medicine (CAM) therapies in treating chronic neck pain, although the evidence is scarce. This study aims to estimate the efficacy and safety of ETN therapy for chronic neck pain.

**Methods and Analysis:**

This is a sham-controlled, randomized clinical trial. A total of 180 subjects will be randomly allocated to either the ETN group or the sham ETN group. Treatment will be administrated three times a week for four consecutive weeks, with a 6-month follow-up. The primary outcome measure will be the Numerical Rating Scale for neck pain (NRS-NP) over a period of the 4 weeks. Secondary outcome measures include the Northwick Park Neck Pain Questionnaire (NPQ), Neck Disability Index (NDI), Patient Global Impression of Change (PGIC), patient expectation, and preference assessment. The chi-square test or Fisher's exact test will be used for proportions of participants having clinically meaningful improvement. Analysis of covariance or repeated-measures analysis of variance will be applied to examine changes in the outcome measures from baseline.

**Discussions:**

This prospective trial will contribute to evaluating the efficacy and safety of ETN in the treatment of chronic neck pain, with an intermediate-term follow-up. This study will provide further evidence for clinical neck pain management.

**Ethics and Dissemination:**

This trial has been approved by the Research Ethical Committee of Guang'anmen Hospital (ethical approval number: 2021-039-KY-01). Recruitment began in March 2022 and will continue until December 2023. Dissemination plans include posters, WeChat, websites, and bulletin boards in hospital and communities.

**Clinical Trial Registration:**

This trial is registered at ClinicalTrials.gov (identifier: NCT04981171).

## Introduction

Chronic neck pain is a common physical complaint across the world and the fourth leading impairment in terms of years lived with disability (1, 2). Its lifetime prevalence is around 48.5% (3). Neck pain is mainly due to poor posture or anxiety, especially in women and the middle-aged population (4). Previous clinical trials have demonstrated that therapies including exercise, acupuncture, and transcutaneous electrical nerve stimulation (TENS) have moderate effects in pain alleviation (5–8). However, management of chronic neck pain remains complex and challenging.

Acupuncture has been widely used for pain alleviating around the world. In previous studies, acupuncture or electroacupuncture is more effective than sham controls (6, 8–11). Clarified evidence supported that acupuncture could produce opioids and other bioactive antalgic chemicals (12). It is thus recommended for the treatment of chronic neck pain by consensus (6, 8, 13). Nevertheless, the application of acupuncture is limited to a large extent due to the patients' fright of and intolerance to needling pain. TENS, a non-invasive physical therapy, is more popular and comfortable. Evidence from clinical trials supported the effectiveness of TENS for chronic neck pain (14–16). The pain could be alleviated *via* stimulation either on acupoints or trigger points, both immediately after treatment and during follow-up periods (15, 17, 18). However, the difference between TENS and sham control is not significant, especially at the long-term follow-ups (14, 18).

A combination of acupuncture with conservational treatment, such as TENS, is considered to benefit the patients more in comparison with single therapy (8, 13). The emergence of electro-thumbtack needle (ETN) therapy is in response to this recommendation, aiming to achieve better effectiveness while maintaining comfortable. The device of ETN is composed of a thumbtack needle with 2 mm long body and a small rechargeable and portable electric stimulator, which outputs current. The surface of thumbtack needle is modified with conductive material to reinforce electric stimulation on acupoints. It is a convenient therapy that can be easily operated by patients on their own after training. In light of the good effectiveness of acupuncture and TENS published in previous studies, as well as the convenience of ETN, we plan to conduct this study to estimate the efficacy and safety of ETN therapy for chronic neck pain in comparison with sham ETN therapy.

## Methods and Analysis

### Study Design

**Figure 1:**
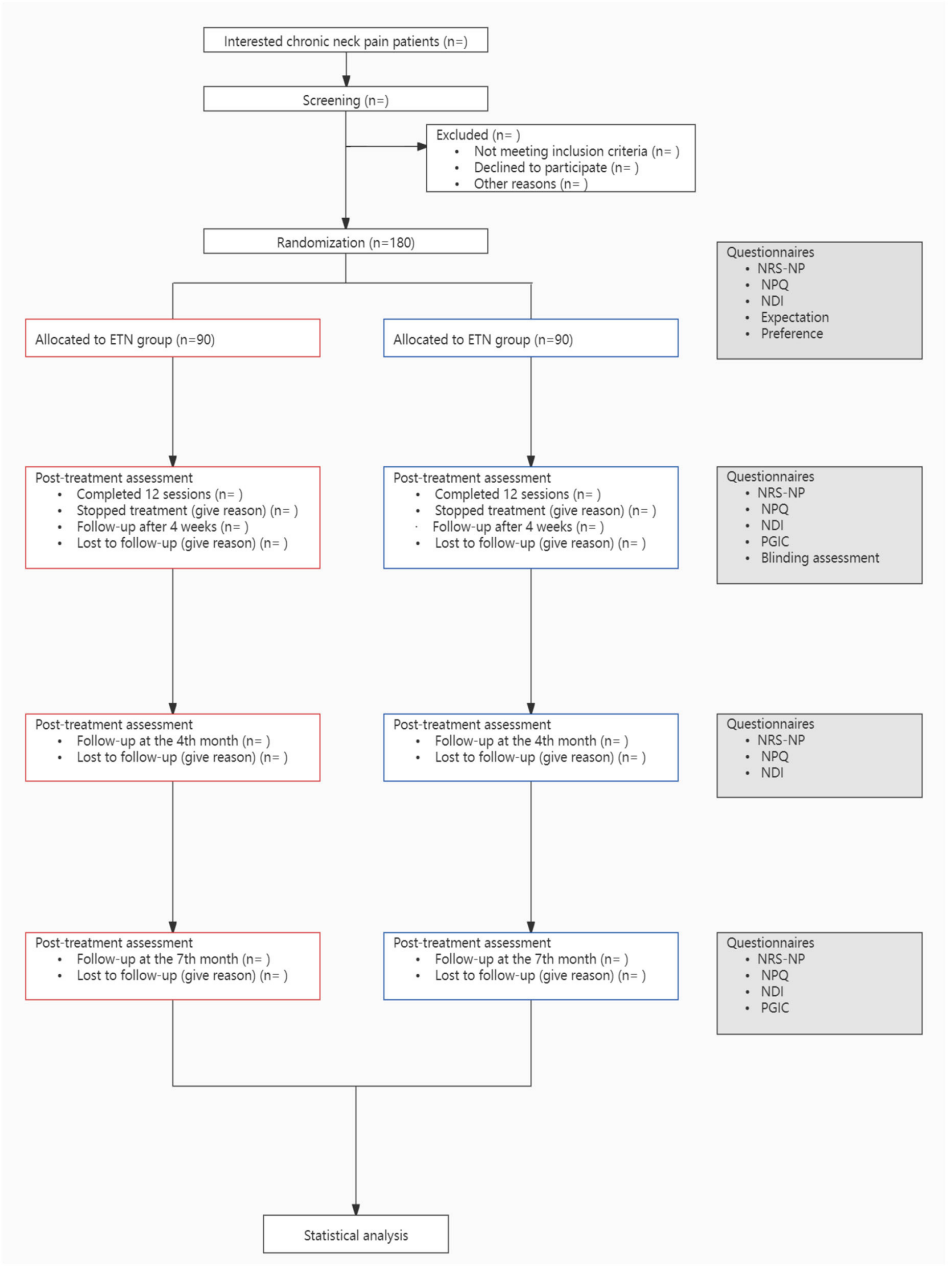
Study flowchart. ETN, electro-thumbtack needle; NRS-NP, numerical rating scale for neck pain; NPQ, the northwick park neck pain questionnaire; NDI, neck disability index; PGIC, patient global impression of change.

This study is a single-center, participant-blinded, randomized controlled trial, which will be conducted from March 2022 to December 2023 in Guang'anmen Hospital, China Academy of Chinese Medical Sciences. The study flowchart is shown in Figure 1. We design this protocol conforming to the guidelines of the Standard Protocol Items: Recommendations for Interventional Trials (SPIRIT) (19) (Table 1) and the Consolidated Standards of Reporting Trials (CONSORT). This trial has been approved by the Institutional Review Board of Guang'anmen Hospital, China Academy of Chinese Medical Sciences (ethical approval number: 2021-039-KY-01), and it has been registered at ClinicalTrials.gov on July 18, 2021 (Identifier: NCT04981171). Each participant will receive 4-week treatment and will be followed up for 6 months.

**Table 1:** SPIRIT 2013 checklist: recommended items to address in a clinical trial protocol and related documents^*^.

**Section/item**	**Item no**	**Description**	**Yes/No**
**Administrative information**			
Title	1	Descriptive title identifying the study design, population, interventions, and if applicable, trial acronym	Yes
Trial registration	2a	Trial identifier and registry name. If not yet registered, name of intended registry	Yes
	2b	All items from the World Health Organization Trial Registration Data Set	Yes
Protocol version	3	Date and version identifier	Yes
Funding	4	Sources and types of financial, material, and other support	Yes
Roles and responsibilities	5a	Names, affiliations, and roles of protocol contributors	Yes
	5b	Name and contact information for the trial sponsor	Yes
	5c	Role of study sponsor and funders, if any, in study design; collection, management, analysis, and interpretation of data; writing of the report; and the decision to submit the report for publication, including whether they will have ultimate authority over any of these activities	Yes
	5d	Composition, roles, and responsibilities of the coordinating center, steering committee, endpoint adjudication committee, data management team, and other individuals or groups overseeing the trial, if applicable (see Item 21a for data monitoring committee)	Yes
**Introduction**			
Background and rationale	6a	Description of research question and justification for undertaking the trial, including summary of relevant studies (published and unpublished) examining benefits and harms for each intervention	Yes
	6b	Explanation for choice of comparators	Yes
Objectives	7	Specific objectives or hypotheses	Yes
Trial design	8	Description of trial design including type of trial (e.g., parallel group, crossover, factorial, single group), allocation ratio, and framework (e.g., superiority, equivalence, non-inferiority, exploratory)	Yes
**Methods: participants, interventions, and outcomes**			
Study setting	9	Description of study settings (e.g., community clinic, academic hospital) and list of countries where data will be collected. Reference to where list of study sites can be obtained	Yes
Eligibility criteria	10	Inclusion and exclusion criteria for participants. If applicable, eligibility criteria for study centers and individuals who will perform the interventions (e.g., surgeons, psychotherapists)	Yes
Interventions	11a	Interventions for each group with sufficient detail to allow replication, including how and when they will be administered	Yes
Interventions: modifications	11b	Criteria for discontinuing or modifying allocated interventions for a given trial participant (e.g., drug dose change in response to harms, participant request, or improving/worsening disease)	Yes
Interventions: adherence	11c	Strategies to improve adherence to intervention protocols, and any procedures for monitoring adherence (e.g., drug tablet return; laboratory tests)	Yes
Interventions: concomitant care	11d	Relevant concomitant care and interventions that are permitted or prohibited during the trial	Yes
Outcomes	12	Primary, secondary, and other outcomes, including the specific measurement variable (e.g., systolic blood pressure), analysis metric (e.g., change from baseline, final value, time to event), method of aggregation (e.g., median, proportion), and time point for each outcome. Explanation of the clinical relevance of chosen efficacy and harm outcomes is strongly recommended	Yes
Participant timeline	13	Time schedule of enrolment, interventions (including any run-ins and washouts), assessments, and visits for participants. A schematic diagram is highly recommended (see Figure 1)	Yes
Sample size	14	Estimated number of participants needed to achieve study objectives and how it was determined, including clinical and statistical assumptions supporting any sample size calculations	Yes
Recruitment	15	Strategies for achieving adequate participant enrolment to reach target sample size	Yes
**Methods: assignment of interventions (for controlled trials)**			
Allocation:			
Sequence generation	16a	Method of generating the allocation sequence (e.g., computer-generated random numbers), and list of any factors for stratification. To reduce predictability of a random sequence, details of any planned restriction (e.g., blocking) should be provided in a separate document that is unavailable to those who enroll participants or assign interventions	Yes
Allocation concealment mechanism	16b	Mechanism of implementing the allocation sequence (e.g., central telephone; sequentially numbered, opaque, sealed envelopes), describing any steps to conceal the sequence until interventions are assigned	Yes
Implementation	16c	Who will generate the allocation sequence, who will enroll participants, and who will assign participants to interventions	Yes
Blinding (masking)	17a	Who will be blinded after assignment to interventions (e.g., trial participants, care providers, outcome assessors, data analysts), and how	Yes
	17b	If blinded, circumstances under which unblinding is permissible, and procedure for revealing a participant's allocated intervention during the trial	Yes
**Methods: data collection, management, and analysis**			
Data collection methods	18a	Plans for assessment and collection of outcome, baseline, and other trial data, including any related processes to promote data quality (e.g., duplicate measurements, training of assessors) and a description of study instruments (e.g., questionnaires, laboratory tests) along with their reliability and validity, if known. Reference to where data collection forms can be found, if not in the protocol	Yes
	18b	Plans to promote participant retention and complete follow-up, including list of any outcome data to be collected for participants who discontinue or deviate from intervention protocols	Yes
Data management	19	Plans for data entry, coding, security, and storage, including any related processes to promote data quality (e.g., double data entry; range checks for data values). Reference to where details of data management procedures can be found, if not in the protocol	Yes
Statistical methods	20a	Statistical methods for analyzing primary and secondary outcomes. Reference to where other details of the statistical analysis plan can be found, if not in the protocol	Yes
	20b	Methods for any additional analyses (e.g., subgroup and adjusted analyses)	No
	20c	Definition of analysis population relating to protocol non-adherence (e.g., as randomized analysis), and any statistical methods to handle missing data (e.g., multiple imputation)	Yes
**Methods: monitoring**			
Data monitoring	21a	Composition of data monitoring committee (DMC); summary of its role and reporting structure; statement of whether it is independent from the sponsor and competing interests; and reference to where further details about its charter can be found, if not in the protocol. Alternatively, an explanation of why a DMC is not needed	Yes
	21b	Description of any interim analyses and stopping guidelines, including who will have access to these interim results and make the final decision to terminate the trial	Yes
Harms	22	Plans for collecting, assessing, reporting, and managing solicited and spontaneously reported adverse events and other unintended effects of trial interventions or trial conduct	Yes
Auditing	23	Frequency and procedures for auditing trial conduct, if any, and whether the process will be independent from investigators and the sponsor	Yes
**Ethics and dissemination**			
Research ethics approval	24	Plans for seeking research ethics committee/institutional review board (REC/IRB) approval	Yes
Protocol amendments	25	Plans for communicating important protocol modifications (e.g., changes to eligibility criteria, outcomes, analyses) to relevant parties (e.g., investigators, REC/IRBs, trial participants, trial registries, journals, regulators)	Yes
Consent or assent	26a	Who will obtain informed consent or assent from potential trial participants or authorized surrogates, and how (see Item 32)	Yes
	26b	Additional consent provisions for collection and use of participant data and biological specimens in ancillary studies, if applicable	No
Confidentiality	27	How personal information about potential and enrolled participants will be collected, shared, and maintained in order to protect confidentiality before, during, and after the trial	Yes
Declaration of interests	28	Financial and other competing interests for principal investigators for the overall trial and each study site	Yes
Access to data	29	Statement of who will have access to the final trial dataset, and disclosure of contractual agreements that limit such access for investigators	Yes
Ancillary and post-trial care	30	Provisions, if any, for ancillary and post-trial care, and for compensation to those who suffer harm from trial participation	Yes
Dissemination policy	31a	Plans for investigators and sponsor to communicate trial results to participants, healthcare professionals, the public, and other relevant groups (e.g., *via* publication, reporting in results databases, or other data sharing arrangements), including any publication restrictions	Yes
	31b	Authorship eligibility guidelines and any intended use of professional writers	Yes
	31c	Plans, if any, for granting public access to the full protocol, participant-level dataset, and statistical code	No
**Appendices**			
Informed consent materials	32	Model consent form and other related documentation given to participants and authorized surrogates	Yes
Biological specimens	33	Plans for collection, laboratory evaluation, and storage of biological specimens for genetic or molecular analysis in the current trial and for future use in ancillary studies, if applicable	No

* *It is strongly recommended that this checklist be read in conjunction with the SPIRIT 2013 Explanation & Elaboration for important clarification on the items. Amendments to the protocol should be tracked and dated. The SPIRIT checklist is copyrighted by the SPIRIT Group under the Creative Commons “Attribution-NonCommercial-NoDerivs 3.0 Unported” license*.

### Eligibility Criteria

#### Inclusion Criteria

Participants conforming to the following criteria are included:

1. Neck pain accompanied with headaches or movement coordination impairments according to the Orthopedic Section of the American Physical Therapy Association (APTA) (7);
2. Aged 18–65 years;
3. History of neck pain for at least 3 months;
4. Scoring at least 4 on Numerical Rating Scale for Neck Pain (NRS-NP) assessing the average neck pain intensity over the last 7 days (12, 20–23).

#### Exclusion Criteria

Participants who meet any of the following criteria are excluded:

1. Neck pain with mobility deficits or radiating neck pain according to the Orthopedic Section, APTA (7);
2. Neck pain secondary to specific diseases, such as tumor, immune disease, endocrine and metabolic disorders, neurological abnormalities, cervical vertebra fracture, and cervical dislocation;
3. Acute or radiating neck pain or pain accompanied with upper limb symptoms;
4. Neck pain with sensory or motor disturbance;
5. Prior cervical spine surgery or congenital abnormalities;
6. Experiencing medical dispute litigation;
7. With the experience of acupuncture in the past 30 days;
8. In the administration of analgesic, muscle relaxant, hormones, or having greater pain in other regions of the body;
9. Allergic to metal or the adhesive tape, implantation of cardiac pacemaker, or ulceration of skin at selected acupoints;
10. Disable to communicate or critically ill;
11. Drug or alcohol dependent;
12. Currently or planning to be pregnant.

### Recruitment

A total of 180 participants with chronic neck pain will be recruited in Guang'anmen Hospital *via* posters, WeChat, websites, and bulletin boards in hospital and communities. Diagnosis will be made by an orthopedist. Eligible participants will be officially enrolled only after signing a written informed consent, and all participants have the right to withdraw their consent later at any time.

### Randomization and Allocation

Eligible participants will be randomly assigned in a 1:1 ratio to either the ETN group or the sham ETN group. The randomization sequence will be generated using a fixed block size by an independent investigator through the SAS 9.4 (SAS Institute, Cary, NC, USA). The randomization number will be sealed in opaque and sequentially labeled envelopes, with the name of the investigator and the date signed over the seals. The envelop will be opened sequentially after the enrollment of participant, and the group allocation will be only informed to the acupuncturist before treatment.

### Blinding

In this trial, the participants, outcome assessors, and the statisticians will all be blinded. The sham ETN was designed to mimic true ETN: the sham thumbtack needle is 0.2 mm long with a blunt tip (instead of a sharp tip), and the electric stimulation is minimal and transient. They will produce a similar sensation of tingling as true ETN, which makes it hard for participants to distinguish. To avoid conversation between groups, participants will be treated separately on alternate days by appointments. After either session in the last week of treatment, blinding assessment will be completed by all participants. They will be told before group allocation: you may be randomly allocated to either the ETN group or the sham ETN group; the electric stimulation is weak in both groups, and you may even be incapable to sense it during the process out of body adaption. Participants will be asked “Which therapy do you think was provided to you?” The available answers include “ETN therapy,” “sham ETN therapy,” or “unclear.”

### Interventions

#### ETN Group

**Figure 2:**
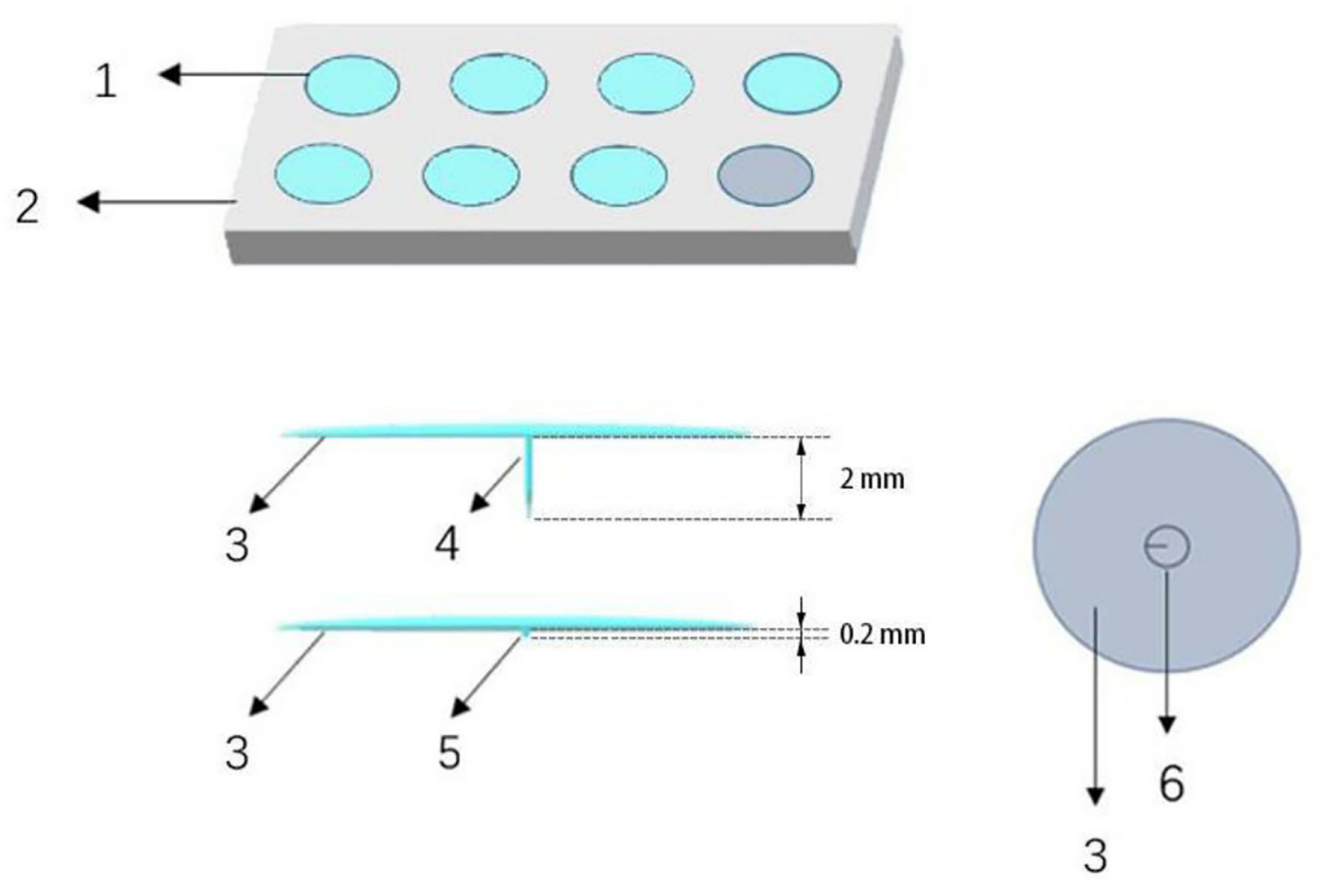
Electro-thumbtack needle and sham thumbtack needle. (1) Thumbtack needle or sham needle, (2) plastic plate, (3) adhesive tape, (4) needle, (5) blunt tip, (6) circular handle.

Thumbtack needle is in the shape of a thumbtack. It has a short body and a circular handle buried in a piece of round medical adhesive tape (Figure 2), which was produced by conductive material. In this trial, thumbtack needles of 0.25 × 2 mm size (Zhenxing, Hangzhou, China) will be used. Acupoints of Dazhui (GV14), bilateral Wangu (GB1 2), bilateral Jiaji at C4 and C6 levels, two Ashi points, and bilateral Houxi (SI3) will be selected in accordance with the theory of traditional Chinese medicine and the consensus of experts.

**Figure 3:**
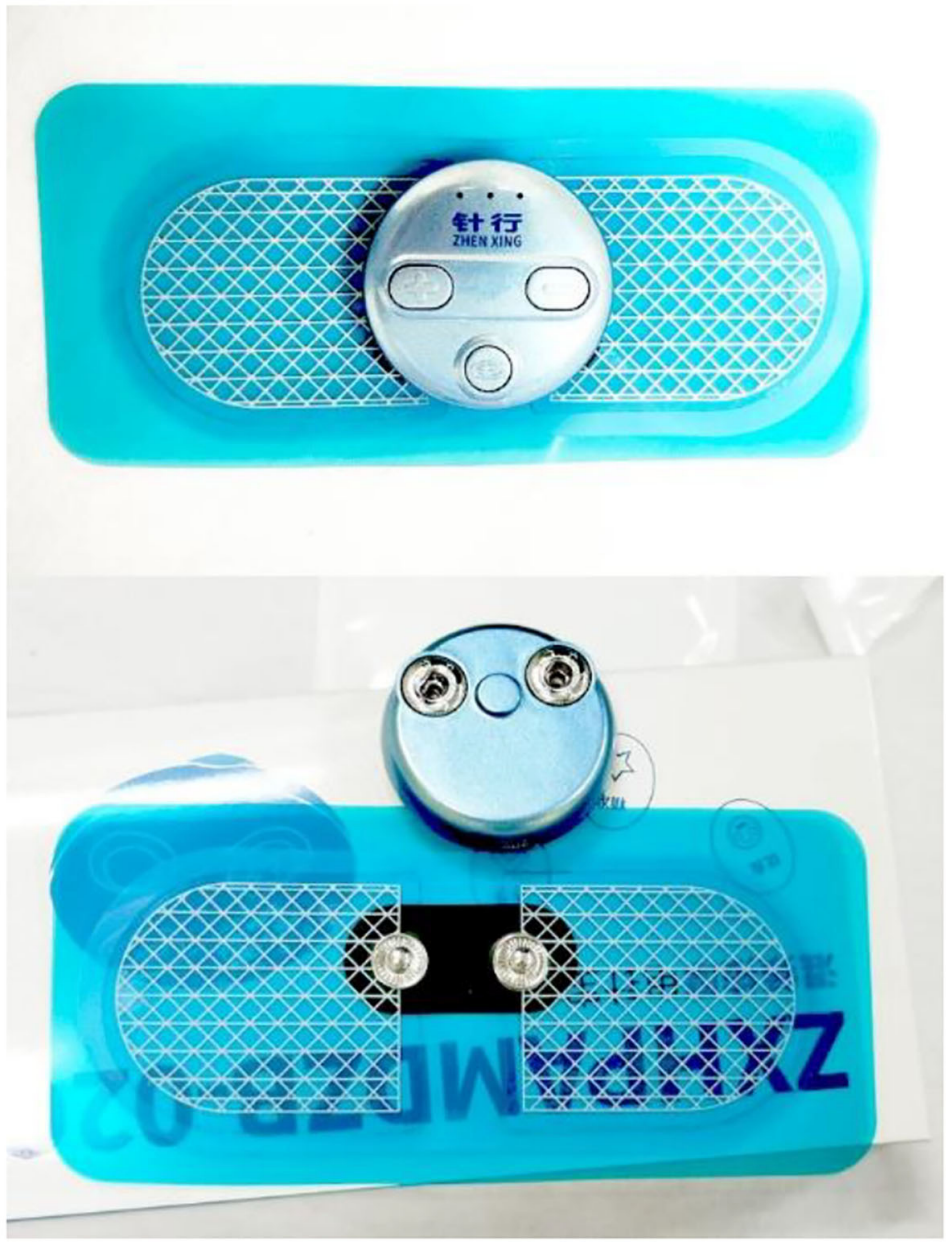
Electric stimulation device and gel electrode.

After sterilizing the skin around the acupoints, the acupuncturist will hold the handle of the needle between the thumb and forefinger, vertically insert the needle into the skin instantly, and adhere medical adhesive tape to skin. After that, the acupuncturist will setup the gel electrodes and the portable stimulation devices (Zhenxing, Hangzhou, China) (Figure 3) to the adhesive tape of all thumbtack needles, switch the mode to low-frequency and discontinuous wave, and adjust the current intensity from level 1 to an appropriate level, which the participant can tolerate. The acupuncturist will gently remove the medical adhesive tape and needles in 30 min.

Participants will receive three 30-min treatment sessions per week (ideally every other day) for 4 consecutive weeks after baseline assessment.

#### Sham ETN Group

We will use sham thumbtack needle specially designed to mimic the true needling. The sham thumbtack needle is 0.2 mm in length and has a blunt tip (rather than sharp) at the bottom. Other than these differences, the conductive surface and other structure of sham thumbtack needle are totally the same as true needle (Figure 2). In the sham ETN group, the blunt tip of needle will stay on the surface and not penetrate the skin, which avoids treatment effect to the largest extent and confuses the participants meanwhile.

**Figure 4:**
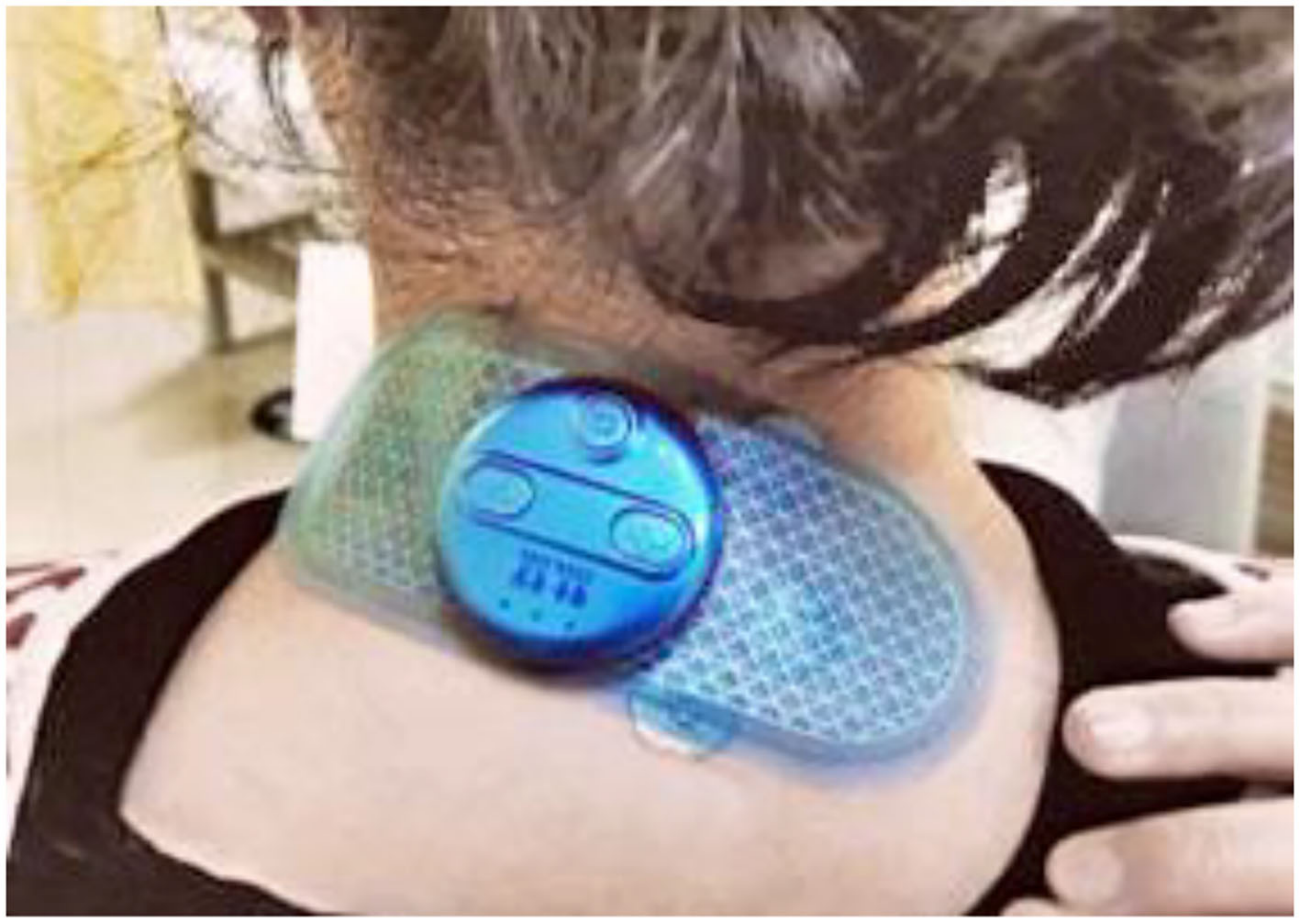
ETN therapy or sham ETN therapy.

Acupoints used in the sham ETN group are the same as those in the ETN group. Acupuncturist will first sterilize the areas of acupoints and then apply the sham needles on the surface of the skin. After assembling the same gel electrodes and portable stimulation devices as in the ETN group (Figures 3, 4), the acupuncturist will adjust the current intensity to level 1 (i.e., the minimum level). The minimal stimulus will last for only 30 s, after which the device will be turned down to cutoff the electric current. The duration of each session will also be 30 min. The frequency of treatment will be all the same as that in the ETN group.

#### Neck Exercises

**Figure 5:**
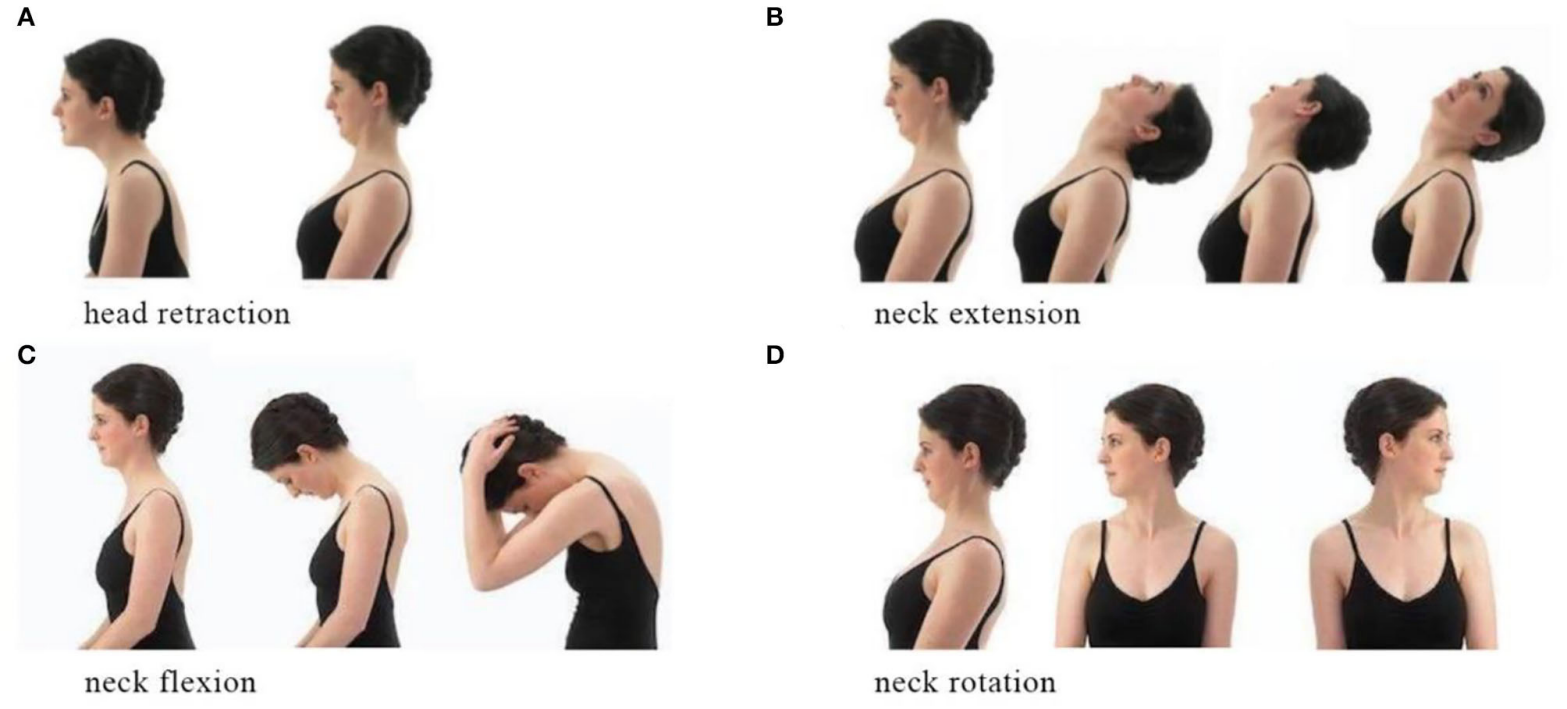
Neck exercise. **(A)** Head retarction. **(B)** Neck extension. **(C)** Neck flexion. **(D)** Neck rotation.

Standardized neck exercise of stretching and strengthening can help participants to relax the neck and acknowledge the appropriate posture. The exercise program consists of head retraction, neck extension, neck flexion, and rotation (Figure 5) (24). Each stretch must reach the maximum angle as possible and be hold for 5–10 s with a 10-s interval (15). When a participant is undergoing severe neck pain, he/she could conduct the exercise in clinostatism (24).

All participants will receive standardized neck exercise instruction at their first treatment session. The neck exercise will be performed at home later. A handbook with detailed illustrations to the exercise will be distributed to participants for guidance. Participants will be asked to perform the standardized exercise one time every day (20) (preferably in the morning) throughout the 4-week treatment. An exercise diary will also be distributed for supervision.

#### Concomitant Treatment

Any other therapy will be discouraged during this trial. Acetaminophen (sustained release type, 500 mg/T) will be provided as a rescue medication with the largest dose of 4 tablets per day for no more than 3 days in total in the condition of unbearable pain. Rescue medication or any other unexpected intervention will be recorded in detail. Proportion of participants and the total days taking rescue medication will be calculated and properly analyzed.

### Outcome Measures

#### Primary Outcome and Measurement

The primary outcome of this trial will be the proportion of participants with at least 50% decrease from baseline in the score of NRS-NP at the 4th week (25). The NRS-NP is a valid and reliable 11-point rating scale to assess the average intensity of neck pain during the last 3 days (26, 27). It is sensitive to small changes with a lower failure rate and can be delivered either graphically or verbally (26–28). The score of NRS-NP ranges from 0 (i.e., no pain at all) to 10 (i.e., the worst pain imaginable). Participants will be asked to circle out 1 of the 11 numbers to describe their pain. The scores of NRS-NP can be categorized into four degrees: 0 (i.e., no pain), 1–3 (i.e., mild), 4–6 (i.e., moderate), and 7–10 (i.e., severe). The reduction of 50% from baseline is regarded as golden standard of the NRS-NP to interpret significant clinically improvement (15, 28, 29).

#### Secondary Outcomes

Secondary outcomes include the proportion of participants with an at least 50% decrease from baseline in NRS-NP score at the 16th and 28th weeks; the changes from baseline in NRS-NP score at the 4th, 16th, and 28th weeks; and the proportion of participants with an at least 30% decrease from baseline in NRS-NP at the 4th, 16th, and 28th weeks. Other secondary outcomes include:

1. Changes in the Northwick Park Neck Pain Questionnaire (NPQ) percentage score from the baseline at the 4th, 16th, and 28th weeks.
2. The proportion of participants with an at least 25% decrease in NPQ percentage score together with a “better” or “much better” at the 4th, 16th, and 28th weeks.

The NPQ is a validated questionnaire to assess the overall symptoms of neck pain (30). It contains 9 questions, the score of each ranging from 0 to 4, on the aspects of intensity, duration, numbness, sleep disturbance, and disability in carrying, watching television, working, social life, and driving. The total score of the 9 questions will be converted to NPQ percentage score (divided by 36 for people who drive and by 32 for those who do not), which ranges from 0 to 100%, and with higher scores indicating worse condition. A 25% decrease in NPQ percentage score and a “better” or “much better” for the 5-scale global effectiveness rating are both needed (31) to reach Minimal Clinically Important Difference (MCID).

(3) Changes from the baseline in Neck Disability Index (NDI) score at the 4th, 16th, and 28th weeks.
(4) The proportion of participants with an at least 10-point decrease from the baseline in NDI score at the 4th, 16th, and 28th weeks.

The NDI is a frequently used instrument to evaluate the self-reporting functional status of patients with neck pain. It consists of 10 items concerning pain, headache, concentration, and daily obligatory activities. Each section is scored with an ordinal scale from 0 to 5, with a maximum score of 50 points (32, 33). Higher scores indicate greater functional limitation. The NDI has been translated into the simplified-Chinese version, which has been shown to have reproducibility, reliability, and validity (34). A 10-point decrease is required to achieve clinically important meaning (27, 35–37).

(5) The proportion of participants reported “much improved” or “very much improved” on Patient Global Impression of Change (PGIC) at the 4th week.

PGIC is a seven-point categorical scale to assess the participant's subjective satisfaction of the treatment (38). It is considered the most reliable instrument for trials concerning chronic pain (38–40). Participants are supposed to report their overall conditions in comparison with before the treatment using one of the following options: “very much worse,” “much worse,” “minimally worse,” “no change,” “minimally improved,” “much improved,” and “very much improved.” Those reported “much improved” or “very much improved” are considered responders of the intervention. It reflects not only the benefits of treatment but also the importance of the benefits for participants themselves (41).

(6) Expectation and belief.

Two questions will be elicited to assess the expectation of and belief in ETN, respectively. For expectation, participants will be asked “How do you think your neck pain will be in 4 weeks?” with the optional of “worse,” “unchanged,” “no idea,” “better,” and “much better.” For belief in ETN therapy, they will be asked “Do you think ETN therapy will be effective in treating chronic neck pain?” with the option of “not effective,” “little effective,” “not sure,” “effective,” and “very effective.” These questions will be elicited at the baseline.

#### Safety Assessment

Acupuncture-associated side effects such as bruising, dizziness, nausea, hematomas, infection, palpitations, or severe pricking pain [visual analog score (VAS) ≥ 4], and all other adverse events (AEs) irrelevant to acupuncture, will be carefully observed and recorded. Serious AEs (SAEs) will be reported to the Medical Ethics Committee of Guang'anmen Hospital within 24 h. After each session, the acupuncturist and outcome assessors will document adverse events and side effects if happens.

### Data Management and Quality Control

The outcome assessor will fill the initial data in case report form and then, input the data into an excel template for data collecting. Two inspectors will check and make sure that all initial data have been correctly input. Any deviation from protocol will be reported to ZL, who will make the final decision if we have to change the protocol or terminate the trial.

Both ETN therapy and sham ETN therapy are safe for participants recruited in our trial; therefore, no intervention modification is permitted, and no unblinding during the trial is permissible. Group allocation will be revealed after the completion of statistical analysis. Participants in the sham ETN group will receive 12 supplementary sessions of ETN therapy if they want. The follow-ups can be conveniently finished by filling in a questionnaire in the clinic, over telephone or through WeChat. This approach could probably improve adherence and make it possible for those who have to discontinue the treatment to complete follow-ups.

### Sample Size

The sample size calculation was based on the primary outcome of the proportion of participants with an at least 50% decrease from the baseline in average neck pain measured by NRS-NP at the 4th week. According to the previous literature, the response rate of TENS plus neck exercise for 4 weeks was reported to be 50% (15); therefore, we presume a 60% response rate of ETN plus neck exercise. For sham ETN plus neck exercise, we presume the response rate to be 38%. Accordingly, a two-tailed testing with an alpha of 0.05, a desired power set at 0.8, and an allowing dropout rate of 20% resulted in 90 participants needed in each group.

### Statistical Analysis

Data calculation will be performed using the SPSS software Ver.20.0 under the ITT principle. Baseline data before intervention will be analyzed to see if the two groups are comparable. The continuous data will be represented as mean ± SD deviation or median [interquartile range (IQR)]. Proportions of participants having clinically meaningful improvement will be analyzed using chi-square test or Fisher's exact test. Changes in the outcome measures from the baseline will be examined by using analysis of covariance for normally distributed data to exclude the possible impact on the effect by covariates of gender and work habits. Repeated-measures analysis of variance will be used if the data are not normally distributed. The Cochran-Mantel-Haenszel test will be used to analyze categorical data, which will be represented by percentages and case. We will use linear regression to discuss the association of participants' expectations and the treatment. Missing research data will be imputed by multiple imputations. For all statistical calculation, the significance level will be two-sided and set at 0.05. A *p* < 0.05 will be considered to indicate statistical significance.

## Discussion

The objective of this study is to evaluate the efficacy and safety of ETN therapy for non-specific chronic neck pain. Demographic characteristics will be collected including age, duration, job, psychosocial or physical risk factors, level of exercise, history of injury, and history of autoimmune disease. The outcomes are designed in accordance with the Initiative on Methods, Measurement, and Pain Assessment in Clinical Trials (IMMPACT) recommendations for core outcomes of chronic pain (41). Both the reduction of 30 and 50% on NRS are applied as outcome measures in this trial. Although the reduction of 30% from baseline in NRS is consistently acknowledged as MCID in current studies (15, 23, 25, 27, 28), a 50% reduction is widely used in clinical trials as a rigorous outcome measurement interpreting more significant improvement in symptoms (15, 28, 29). Additionally, multiple outcomes will be evaluated, as various as pain intensity, disability, quality of life, and the degree of subjective satisfaction.

The effectiveness of both manual acupuncture and TENS has been identified by previous studies (15, 42) in reducing chronic neck pain. Since ETN treatment has been an integration of thumbtack needling and electric stimulation, the treatment effect will probably be stronger and comprehensive. Notably, it is easy to administrate thumbtack needles on acupoints, even by patients themselves at home.

In this trial, blunt-tip thumbtack needle with no penetration and transient minimal electric stimulation is designed as sham control. It can produce acupuncture-like sensation of tingling or pain by pressing the blunt tip on acupoints without skin penetrating, similar to the validated placebo needle (43, 44). To maintain the success of blindness, participants from different groups will be avoided to contact with each other. The outcome assessor and the statistician will also be concealed to group allocation. These approaches are constructed to reduce possible biases attached to the results.

Limitations of this study are as follows. Group allocation will be not blinded to the acupuncturist, whose attitude for or against an intervention might be inevitably transferred to participants. Besides, this study does not implement a waitlist control in consideration of ethics principles, in which condition we cannot exclude the influence of spontaneous remission of disease.

This prospective trial will evaluate the efficacy and safety of ETN for chronic neck pain and provide evidence for clinical health recommendations.

## Ethics Statement

The studies involving human participants were reviewed and approved by the Research Ethical Committee of Guang'anmen Hospital, China Academy of Chinese Medical Sciences. The patients/participants provided their written informed consent to participate in this study.

## Author Contributions

ZL, HS, and XW designed this trial and drafted the manuscript over discussion. The study protocol was revised by YY, LZ, YC, and SG. HS and XW contributed to the ethical application and the implementation of the trial. All authors approved the publication of this final manuscript.

## Funding

This study is funded by Guang'anmen Hospital, China Academy of Chinese Medical Sciences.

## Conflict of Interest

The authors declare that the research was conducted in the absence of any commercial or financial relationships that could be construed as a potential conflict of interest.

## Publisher's Note

All claims expressed in this article are solely those of the authors and do not necessarily represent those of their affiliated organizations, or those of the publisher, the editors and the reviewers. Any product that may be evaluated in this article, or claim that may be made by its manufacturer, is not guaranteed or endorsed by the publisher.
